# Risk Factors of Coronary Artery Abnormality in Children With Kawasaki Disease: A Systematic Review and Meta-Analysis

**DOI:** 10.3389/fped.2019.00374

**Published:** 2019-09-26

**Authors:** Fan Yan, Bo Pan, Huichao Sun, Jie Tian, Mi Li

**Affiliations:** ^1^Department of Cardiology, Children's Hospital of Chongqing Medical University, Chongqing, China; ^2^Ministry of Education Key Laboratory of Child Development and Disorders, Chongqing, China; ^3^National Clinical Research Center for Child Health and Disorders (Chongqing), Chongqing, China; ^4^Chongqing Key Laboratory of Pediatrics, Chongqing, China; ^5^China International Science and Technology Cooperation Base of Child Development and Critical Disorders, Chongqing, China

**Keywords:** risk factors, coronary artery abnormality, Kawasaki disease, systematic review and meta-analysis, children

## Abstract

While coronary artery abnormality (CAA) has been established as the most serious complication of Kawasaki disease (KD), there have been no detailed systematic reviews of the risk factors associated with this condition. We searched six databases and performed a systematic review and meta-analysis. Study-specific odds ratios (ORs) for each factor were pooled using a random effects model. We identified four risk factors for CAA children with KD: gender (OR, 1.75; 95% confidence interval [CI], 1.59–1.92), intravenous immunoglobulin (IVIG) resistance (OR, 3.43; 95% CI, 2.07–5.67), IVIG treatment beyond 10 days of onset of symptoms (OR, 3.65; 95% CI, 2.23–5.97), and increased C-reactive protein levels (OR, 1.02; 95% CI, 1.01–1.02). More number of the five typical symptoms of KD was identified as a protective factor against CAA (OR, 0.47; 95% CI, 0.33–0.66). Pediatric patients with IVIG resistant were more likely to develop CAA within 1 month of the onset of KD than the general population, even in patients with other risk factors for CAA. Thus, there is a potential risk of CAA misdiagnosis if echocardiography is not carried out frequently. In summary, we report four risk factors for CAA and a protective factor against CAA in children with KD. We recommend that pediatricians consider these factors much more closely. With accurate prediction and early preventive treatment in high-risk patients, we can expect a reduction in CAA rates. Further research is now required to investigate the associations between CAA and other factors including the interval between KD onset and IVIG administration, platelet count, and the duration of fever. We also need to confirm whether the frequency of echocardiography within a month of KD onset should be increased in IVIG-resistant patients.

## Introduction

Kawasaki disease (KD) is an acute self-limited disorder characterized by systemic vasculitis and predominantly occurs in early childhood (1, 2). The etiology of KD remains unknown and there are no specific diagnostic tests. Consequently, KD is characterized by fever in addition to numerous typical physical findings, including bilateral non-exudative conjunctivitis, erythema of the lips and. oral mucosa, changes in extremities, rashes, cervical lymphadenopathy and laboratory evidence of a systemic inflammatory response (3, 4). Coronary artery abnormality (CAA) is the most serious complication occurring in 15–25% of untreated patients and is a persistent highlight in KD research (3)

Intravenous immunoglobulin (IVIG) is widely administered as the initial first-line treatment and some patients with a high risk of CAA are treated with adjunctive therapy such as corticosteroids and infliximab. However, despite these interventions, the reported incidence of CAA rate still exceeds 30% in some literatures. It is therefore very important to determine the potential risk factors of CAA in children with KD. Accordingly, previous research studies created a series of scoring systems to predict IVIG-resistant KD, considered as an important risk factor for CAA and the development of CAA in Japanese patients, such as the Harada, Kobayashi, Sano and Egami scoring systems (5–8). However, these systems were not as sensitive and specific in other populations as they were in the Japanese population (9). Moreover, these systems incorporate too many indicators and have never been systematically reviewed in detail except for a study concerning the incomplete presentation of KD and a meta-analysis investigating CAA risk factors in Chinese children (10, 11). Therefore, we conducted a systematic review and meta-analysis to investigate the risk factors of CAA in children with KD by analyzing the most up-to-date observational studies.

## Materials and Methods

### Study Design

This systematic review and meta-analysis is reported in accordance with the Preferred Reporting Items for Systematic Reviews and Meta-Analyses (PRISMA) Statement (Checklist S1) and was registered in the International Prospective Register of Systematic Reviews (reference number: CRD42018076512).

### Participants

Our analysis represented two groups: the control group—the KD patients without CAA, and the CAA group—the patients with onset of KD and CAA found in a given period of medical follow-up.

### Search Strategy

We conducted a systematic literature search using electronic databases, including PubMed, Embase, Web of Science, Cochrane Library, and the National Institutes of Health Clinical Trial Databases up to the 16th May 2018. We also searched OvidMedline from 1946 to 16th May 2018. The search strategy used Medical Subject Headings (MeSH) terms or Emtree thesaurus terms combined with keywords for [(Mucocutaneous Lymph Node Syndrome) and (Coronary Artery)]. There was no language restriction. In addition, we manually searched the reference lists of original and review articles for further articles of interest. Some texts were unavailable online; in these cases, we attempted to contact the authors via email.

### Inclusion and Exclusion Criteria

Our analysis included observational studies which fulfilled the following criteria: (1) Study participants were children diagnosed with KD, including both CKD and IKD; (2) All participants diagnosed with KD met the specific criteria published by the Japan Kawasaki Disease Research Committee (4th or 5th revised edition) or the American Heart Association (2001, 2004, or 2017 edition); (3) CAA was one of the outcome measures and was detected by ultrasonic cardiography as an existing abnormal body surface area adjusted by z-scores or abnormal internal lumen diameter according to criteria published by the Japanese Ministry of Health, American Heart Association, or by Chinese literature (3, 12); (4) Risk factors for CAA were investigated with no restriction to specific subgroups; (5) The study reported the odds ratio (OR) adjusted for at least one potential confounder and 95% confidence intervals (CIs) or allowed for the calculation of these parameters from the raw data presented in the article and (6) The article was written in English.

We excluded studies that examined risk factors for CAA in animal populations. We also excluded studies that were restricted to a specific clinical subgroup of KD patients, such as IVIG-resistant KD, atypical KD, recurrent KD or KD in pregnant women. We also excluded case reports, case series, reviews, letters, commentaries, conference papers and studies relating to the pathogenesis and genetics of KD.

### Data Extraction and Quality Assessment

For each eligible study, two investigators (Fan Yan and Bo Pan) independently extracted the following information: author names, publication year, study design, follow-up duration (by echocardiography), study duration, study location, sample size, total number of CAA cases, diagnostic criteria used for KD, diagnostic criteria used for CAA, the definition of IVIG-resistant KD, risk factors and the methods used for statistical analysis. Quality assessments were also independently conducted by the two investigators (Fan Yan and Bo Pan) using the Newcastle-Ottawa Quality Assessment Scale for case control or cohort studies (Checklist S2, Supporting Tables 1, 2); disagreements were resolved by group discussions.

### Statistical Analysis

To control confounders, we included studies reporting estimates which adjusted for at least one potential confounder in their analysis; this strategy has been used in previous literature (13). We believe that this is a feasible approach for eliminating some publications with low evidence levels. When a reported risk factor was evaluated by three or more studies, considering the intrinsic differences of study design, we combined the adjusted ORs and 95% CIs with the random effects model to estimate pooled ORs and associated 95% CIs. The I^2^ statistic was used to investigate the heterogeneity across studies; an I^2^ value of <25% and >50% was considered to indicate low and high levels of heterogeneity, respectively (14). We also performed sensitivity analysis to assess the robustness of our results by omitting a single study in turn. We also conducted the Egger regression asymmetry test and the Begg test to detect the presence of publication bias; this analysis showed that male gender was the only factor contributing to publication bias (all other factors were found in fewer than 10 studies). Statistical analyses were performed using STATA version 15 (StataCorp, College Station, TX).

## Results

**Figure 1:**
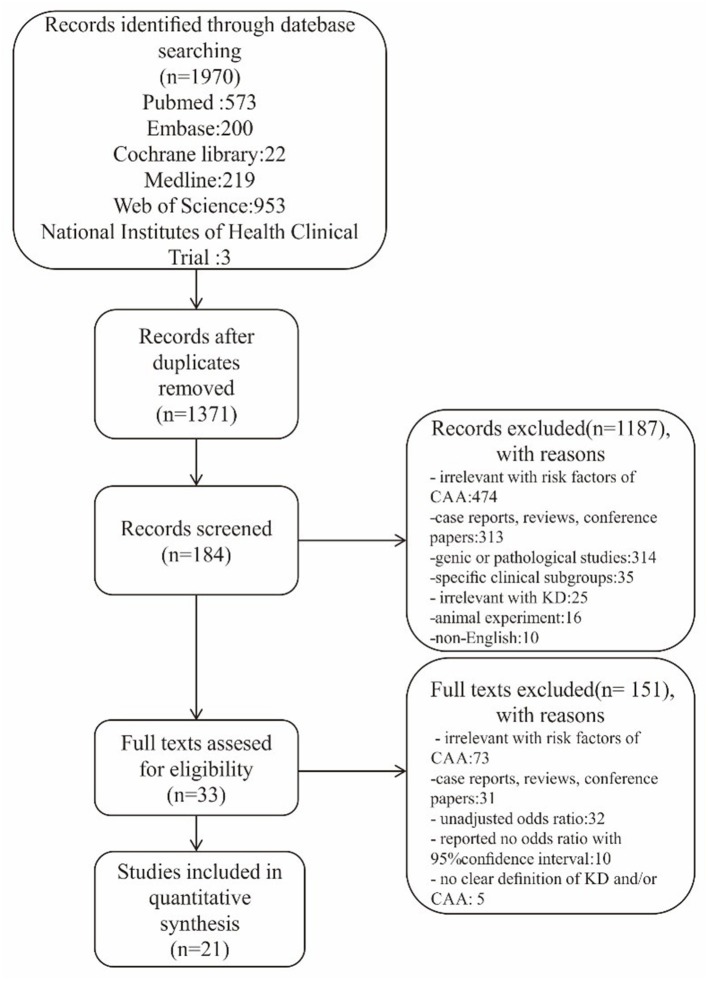
Flow diagram of studies screened in the meta-analysis.

In total, our initial search identified 1,970 articles. After removing duplicates and reviewing the titles and abstracts, 184 studies were potentially eligible for inclusion. After assessing the full text, 33 studies were found to conform to our specific inclusion criteria. Finally, 21 of the 33 studies were included in our meta-analysis; collectively, these studies included 26,684 participants, of which 4,461 were diagnosed with CAA, contributing 9 risk factors investigated in more than two studies (Figure 1). Specific characteristics of these data are summarized in Table 1.

**Table 1:** Summary of the included studies for quantitative synthesis.

**Study**	**Study design**	**Duration of following-up (by echocardiogram)**	**Study duration**	**Study location**	**Population sample size**	**Total number of CAA**	**Diagnostic criteria of KD**	**Diagnostic criteria of CAA**	**Define of IVIG-resistant KD**	**Risk factors**	**Method of statistical analysis**	**NOS score**
Yeo et al. (15)	Case-control	At least 2 months	2001–2006	Korea	136	16	AHA2004	JMH	ND	Days of fever, number of symptoms	Logistic regression analysis	7
Hamza et al. (16)	Case-control	Eight weeks	2012–2016	Egypt	64	34	AHA2017	Z score	ND	Platelet count	Logistic regression analysis	7
Wilder et al. (17)	Case-control	ND	1991–2002	America	324	21	AHA2004	Z score	ND	Diagnosis after illness day 10	Logistic regression analysis	7
Weng et al. (18)	Cohort study	Eight weeks	1993–2009	Taiwan	2,116	81	AHA2004	JMH	Fever for 3 days after initial IVIG	Neutrophil count,Dose of IVIG, platelet count	Logistic regression analysis	7
Tajima et al. (19)	Case-control	1 month	2006–2012	Japan	100	13	JKDRC	JMH	A second Dose IVIG	Delayed IVIG (≥6 days), IVIG-resistant	Logistic regression analysis	7
Song et al. (20)	Case-control	At least 2 months	2001–2007	Korea	221	30	AHA2004	JMH	fever for 2 days after initial IVIG	Number of symptoms, Post-IVIG fever, Harada score	Logistic regression analysis	7
Sabharwal et al. (21)	Case-control	6–8 weeks	1990–2007	Canada	1,374	266	AHA2004	JMH	Absence of fever within 36 h after initial IVIG	Age, male, duration of fever before diagnosis, albumin level, hemoglobin level, platelet count, IVIG-resistant	Logistic regression analysis	8
Ruan et al. (22)	Case-control	1 month	2003–2009	China	1,370	486	AHA2004	JMH	ND	Age (<6 months), Male, Time of IVIG, IVIG dose, platelet count and ESR	Logistic regression analysis	7
Qiu et al. (23)	Cohort study	ND	2009–2014	China	930	179	Similar to JKDRC	Chinese literatures criteria	ND	Treatment time, treatment time 10 days	Logistic regression analysis	7
Patel et al. (24)	Case-control	ND	1994–2008	Denmark	284	37	Similar to AHA2004	AHA	ND	Age, male, time of IVIG (>10 days)	Logistic regression analysis	7
Kim et al. (25)	Case-control	At least 2 months.	2001–2005	Korea	285	19	AHA2001	JMH	ND	Total days of fever >8 days	Logistic regression analysis	7
Young et al. (26)	Cohort study	6–8 weeks	2005–2013	Korea	703	266	AHA2004	Z score	Received more than one dose of IVIG	Male, fever duration (≥8 days), CRP (≥7 mg/dl), WBC count (>12 ×103/μL)	Logistic regression analysis	8
Ghelani et al. (27)	Case-control	ND	2000–2002 and 2007–2009	America	203	33	AHA2004	Z score or JMH	Fever after one dose of IVIG and administration of additional IVIG or use of corticosteroids or TNF-alpha blockers	ESR, refractory Kawasaki disease	Logistic regression analysis	7
Chen et al. (28)	Case-control	1 month	2008–2012	China	2,302	365	JKDRC	JMH	ND	Male, age (≤ 1 year), IVIG unresponsiveness, time of IVIG	Logistic regression analysis	7
Lega et al. (29)	Case-control	6–8 weeks	1988–2007	France	194	64	AHA2004	AHA	Fever ≥36 h after complete IVIG infusion	Male, Age, PE, Hemoglobin level, IVIG resistance	Logistic regression analysis	7
Boudiaf and Achir (30)	Case-control	4–6 weeks	2005–2014	Algeria	133	30	AHA2004	Z score	ND	Duration of fever (>10 days), platelet count (>450,000/mm^3.^)	Logistic regression analysis	8
Berdej-Szczot et al. (9)	Case-control	No clear description	2003–2016	Poland	73	13	AHA	AHA	Fever >36 h after IVIG	Delay diagnosis, platelet count, additional symptom	Logistic regression analysis	8
Kim et al. (31)	Case-control	ND	2012–2014	Korea	5,151	524	AHA2004	JMH	A second dose of IVIG	CRP	Logistic regression analysis	7
Xu et al. (32)	Case-control	3 months	2009–2012	China	422	83	JKDRC	Chinese literatures Criteria	ND	RDW (>14.55%), IVIG resistance,Fever duration (>14 days)	Logistic regression analysis	8
Callinan et al. (33)	Case-control	ND	2000–2009	America	1,843	341	Similar to AHA2004	AHA	ND	Male, age, race, time of IVIG (>5 days)	Logistic regression analysis	7
Kim et al. (34)	Cohort study	3 months	ND	Korea	8,456	1,560	AHA2004	Z score or JMH	Existence of second-line treatment	Male, age, fever duration, incomplete presentation, recurrent illness, high/medium-dose ASA, non-response to first-line treatment, total bilirubin, CRP	Logistic regression analysis	6

*NOS, Newcastle-Ottawa Quality Assessment Scale; AHA, American Heart Association; JMH, Japanese Ministry of Health; ND, no description; Z score, body surface area adjusted z-scores; JKDRC, Japan Kawasaki Disease Research Committee; ESR, erythrocyte sedimentation rate erythrocyte sedimentation rate; PE, pericardial effusion; CRP, C-reactive protein; RWD, red blood cell distribution width*.

### Risk Factors

#### Gender

**Figure 2:**
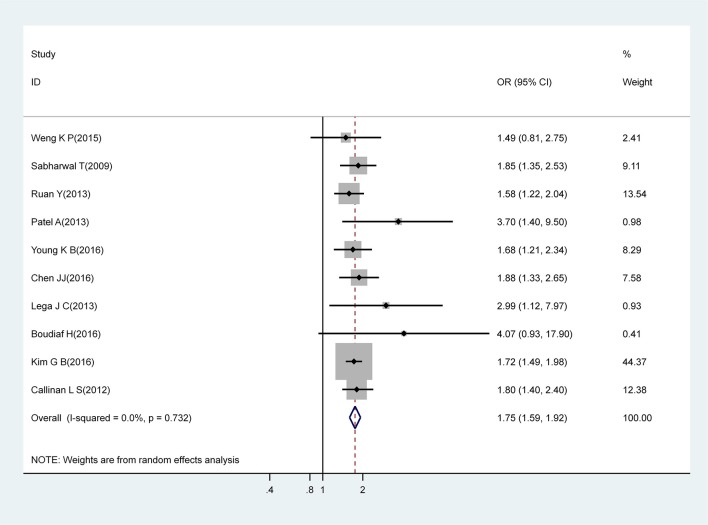
Pooled odds ratio for CAA by gender (male vs. female).

Meta-analysis of 10 studies that estimated the association between male gender and CAA identified that males had a significantly higher risk for CAA (OR, 1.75; 95% CI, 1.59–1.92), with no evidence of heterogeneity (I^2^ = 0%; *P* = 0.732) (Figure 2).

#### IVIG Resistance

**Figure 3:**
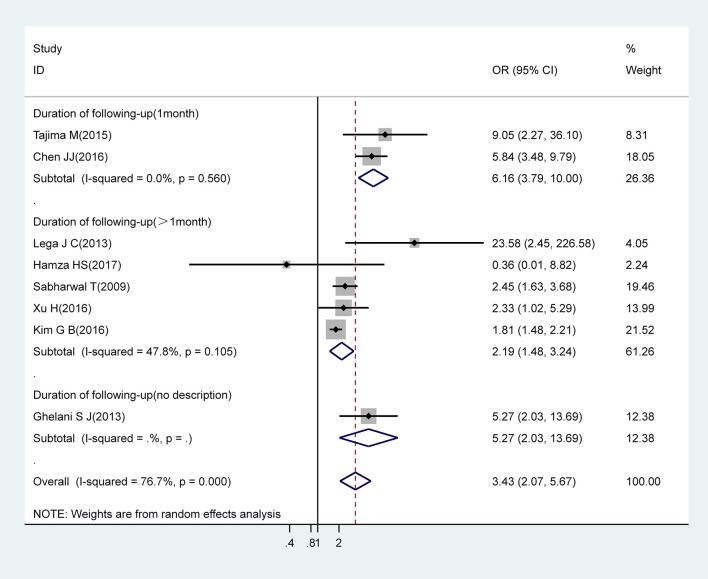
Pooled odds ratio for CAA by IVIG-resistance (subgroup analysis basing on duration of following-up).

Pooled estimates from eight studies revealed that IVIG resistance markedly increased the risk for CAA (OR, 3.43; 95% CI, 2.07–5.67). However, there was significant heterogeneity between these eight studies (I^2^ = 76.7%; *P* = 0.000). Subgroup analysis showed that a follow-up duration of >1 month was an increased risk factor for CAA (OR, 2.19; 95% CI, 1.48–3.24) with acceptable levels of heterogeneity (I^2^ = 47.8%; *P* = 0.105). A follow-up duration of ≤ 1 month was associated with an increased risk for CAA (OR, 6.16; 95% CI, 3.79–10.00) with low levels of heterogeneity (I^2^ = 0%; *P* = 0.560) (Figure 3).

#### IVIG Treatment Beyond 10 Days of Onset of Symptoms

**Figure 4:**
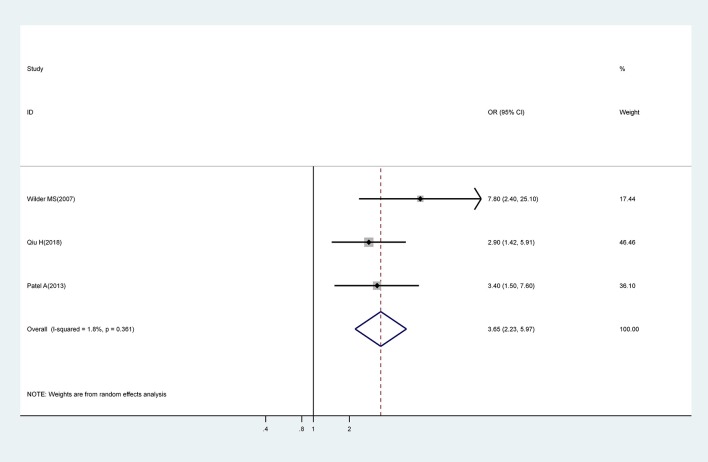
Pooled odds ratio for CAA by time of initial IVIG treatment (IVIG > 10 days vs. IVIG ≤ 10 days).

Meta-analysis of three studies showed that IVIG treatment beyond 10 days of onset of symptoms was associated with a significantly higher risk for CAA (OR, 3.65; 95% CI 2.23–5.97) with low levels of heterogeneity (I^2^ = 1.8%; *P* = 0.361) (Figure 4).

#### C-Reactive Protein (CRP)

**Figure 5:**
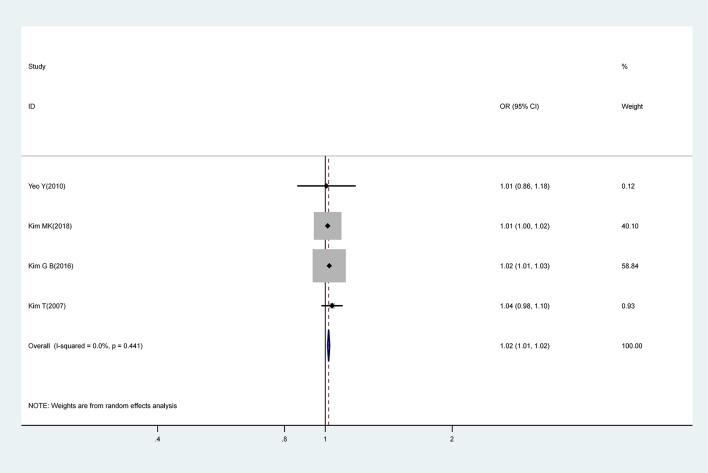
Pooled odds ratio for CAA by CRP (mg/L).

Meta-analysis of pooled estimates from four studies revealed that a 1 mg/L increase in CRP levels was associated with a 0.02-fold increase in risk for CAA (OR, 1.02; 95% CI 1.01–1.02); with low levels of heterogeneity (I^2^ = 0.0%; *P* = 0.441) (Figure 5).

#### The Number of Symptoms

**Figure 6:**
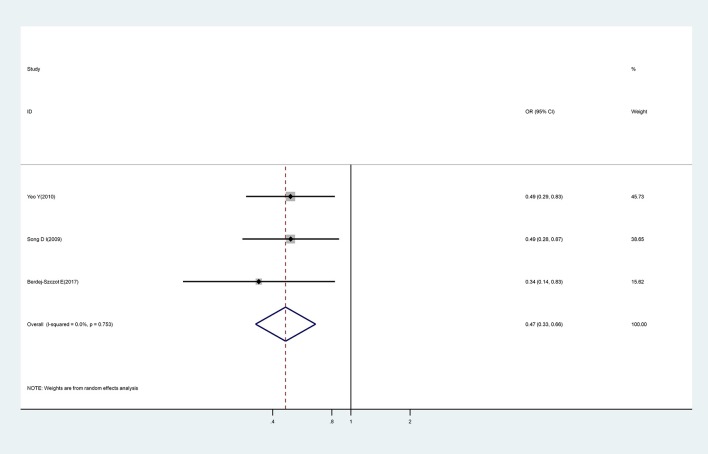
Pooled odds ratio for CAA by number of the five typical symptoms of KD.

We investigated three studies which attempted to identify an association between the number of presenting symptoms and CAA. An increasing number of the five typical symptoms of KD was shown to represent a significant protective factor for CAA (OR, 0.47; 95% CI, 0.33–0.66), with no evidence of heterogeneity (I^2^ = 0.0%; *P* = 0.753) (Figure 6).

#### Other Factors

**Figure 7:**
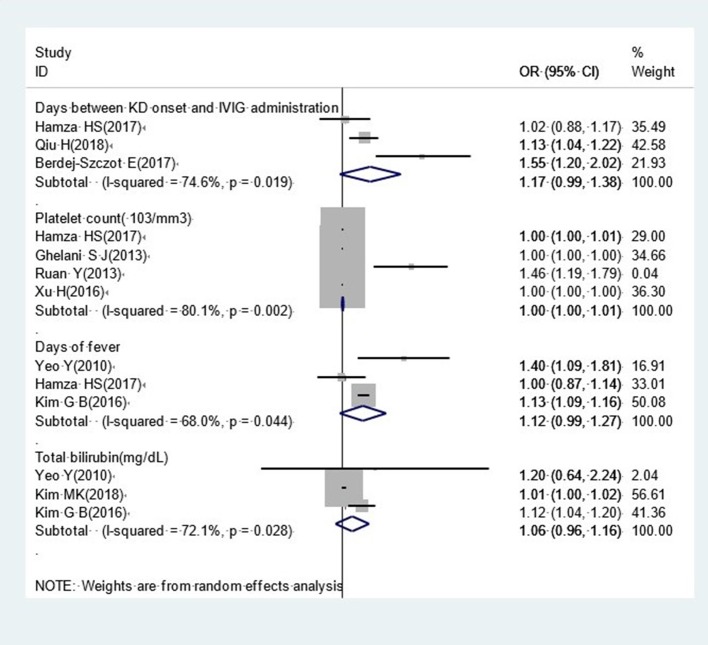
Pooled odds ratios for CAA by other factors.

Our analysis identified several factors that were not significantly associated with CAA, including the interval between KD onset and IVIG administration (OR, 1.17; 95% CI, 0.99–1.38), platelet count (OR, 1.00; 95% CI, 1.00–1.01), the duration of fever (OR, 1.12; 95% CI, 0.99–1.27) and total bilirubin (OR, 1.06; 95% CI, 0.96–1.16) (Figure 7).

## Discussion

CAA is a commonly encountered and serious complication of KD and is considered as a leading cause of acquired heart disease in children. Identifying patients at high risk of developing CAA at an early stage after the onset of KD is important for determining a more intensive treatment, the duration of follow-up and targeted research studies aimed at identifying new therapeutic methods for KD. In this meta-analysis, we identified five factors, based on good evidence that were strongly associated with CAA: male gender, IVIG resistance, IVIG treatment beyond 10 days of onset of symptoms, increased CRP levels, and the number of presenting symptoms. To the best of our knowledge, our study is the first meta-analysis to comprehensively investigate the risk factors associated with CAA. Only two other related studies have been published: one investigated the incomplete presentation of KD as a risk factor for CAA, the other investigated risk factors for CAA in Chinese children but with non-convincing evidence (10, 11).

While previous studies failed to determine an association between CAA and gender (35, 36), we successfully identified male gender as a strong risk factor for CAA; this difference is likely to be due to the increasing number of multicenter studies being reported with large sample sizes. Epidemiological surveys, carried out in different biogeographical regions, also identified higher incidence rates of KD in males, the boys with KD outnumbered the girls with KD by the ratio approximately 1.5–1.7:1 (37, 38). Until now, there is no convincing explanation for this gender bias, although Kobayashi and Dallaire noted that healthy male children tended to have larger coronary arteries than female children (39, 40), thus making the diagnosis of KD and CAA easier in males when using the same diagnostic criteria. There are no obvious differences in terms of estrogen level when compared between genders in children, consequently it is difficult to explain the incidence of CAA in children with KD as an autoimmune disease by estrogen levels alone, as is the case for some autoimmune diseases in adults (41). However, it has been demonstrated that different genders express variable levels of miRNA expression and that these differences may play a role in the immune response and autoimmune diseases (42, 43).

IVIG treatment is a well-established primary treatment measure for KD, and several studies have linked IVIG factors (such as dose and brand) with a higher risk of CAA (18, 44). We found that IVIG resistance and IVIG treatment beyond 10 days of onset of symptoms was associated with an increased risk for CAA. IVIG is considered to alleviate coronary injury by regulating the immune system, including the reduction of cytokine levels and the suppression of endothelial cell activation (45, 46). However, studies have reported IVIG resistance rates as high as 18–22%; accordingly, several scoring systems have been developed to predict IVIG-resistant KD. Because CAA usually occurs 7–10 days after the onset of KD onset (47, 48), the AHA published guidelines in 2004 which stated that “therapy should be instituted within the first 10 days of illness and, if possible, within 7 days of illness.” However, some researchers believe that the status of the illness can potentially exert influence on the association between delayed IVIG treatment and CAA. For example, Qiu observed higher CRP levels and ESR in patients in which IVIG treatment was delayed (23). However, delayed IVIG treatment can cause increased CRP levels and ESR, and there is no good evidence to determine the causal factor between these factors. In contrast, higher CRP levels and ESR represent a more aggressive inflammatory response, causing a more typical manifestation of the illness with advanced intervention by IVIG treatment. Further studies are now required to clarify whether there is a correlation between the status of KD and delayed IVIG treatment.

We also noticed that patients with a 1 month follow-up period showed an obvious increase in the risk of CAA compared with those with a follow-up duration of >1 month among the IVIG-resistant population. AHA guidelines recommend that echocardiographic evaluation is recommended at the time of diagnosis, at 2 weeks, and at 6–8 weeks after the onset of disease. In a previous study, Tajima and Chen conducted echocardiographic examinations before and after IVIG treatment and 1 month after the diagnosis of KD (19, 28); this suggested that echocardiographic evaluations should be carried out more frequently within 1 month of the onset of KD than those recommended by the AHA guidelines. This could explain the differences between subgroups. Based on these findings, we attempted to verify whether this difference also existed in other populations. Unfortunately, with the limited number of studies included for other risk factors, we were only able to carry out the same subgroup analysis in the male population. However, in situations where studies included in our subgroup analysis were somewhat similar to the IVIG-resistant population, we found no noticeable difference in the risk of CAA when compared between shorter and longer follow-up periods (OR, 1.68; 95% CI, 1.37–2.06 vs. OR, 1.74; 95% CI, 1.55–1.96) (Supporting Figure 1). In summary, children with KD who were resistant to IVIG appeared to be more likely to develop CAA within 1 month after KD onset than the general population, even compared to patients with other risk factors for CAA. As a consequence, there are potential risks for the misdiagnosis of CAA when echocardiography is not carried out frequently. Whether echocardiography should be performed more often within 1 month of KD onset in an IVIG-resistant population remains to be confirmed by future studies.

Our meta-analysis found an apparently increased risk of CAA in children with elevated CRP levels. However, this did not appear to be a neglectable risk factor with a magnitude of 10 mg/L increasing frequently. When considered as a categorical variable, as in the present study and many others, an elevated CRP level is an important risk factor of CAA (5, 26, 49). Furthermore, some recent studies that have investigated the association between atherosclerosis and inflammatory status clearly recognized the fact that CRP is also a reliable marker of inflammation and the prediction of coronary events (50–52). However, in another study, Kim studied the genetic loci that influence the levels of CRP and identified a CRP locus that is associated with high CRP levels but without affecting the development of CAA (53). However, this result may have been due to the small sample size and the fact that this previous study focused only upon a single race (Korean); it is also possible that some other underlying cause may influence the development of CAA. Furthermore, one has to consider genotypic and phenotypic diversity and irrelevant factors associated with potential confounding factors, such as gender, the duration of IVIG treatment, and the number of symptoms. One may therefore speculate that determining the primary characteristics of individuals rather than the genetic features of KD is the key in making a specific and sensitive prediction regarding CAA.

Although we did not acquire sufficient studies with which to perform a meta-analysis for the relationship between atypical KD and CAA development, we still identified that more number of the five typical symptoms of KD was a strong protective factor for CAA. However, some previous studies have stated that delays in diagnosis and IVIG treatment are associated with atypical forms of KD (54, 55). Furthermore, children of a younger age have consistently been associated with atypical KD (24, 29). However, in all three of the studies included in our meta-analysis, no significant differences were identified in terms of age and/or interval between KD onset and IVIG administration when compared between CAA(+) and CAA(–) patients. Even after adjusting for such factors, more number of symptoms was clearly a protective factor of CAA. It seems reasonable to suggest that a lower number of symptoms relates to the manifestation of an immature or dysfunctional immune response during the onset of disease.

Although the interval between KD onset and IVIG administration, platelet count, and the duration of fever were not expected to show an obvious relationship with the development of CAA, the lower values of the narrow confidence intervals for these factors were very close to 1.0. Given that we could only include a limited number of studies to investigate these factors, it is possible that our final results could easily be changed by introducing more studies into our meta-analysis. As shown in Figure 7, the study reported Hamza was responsible for negative results in our meta-analysis relating to the interval between KD onset and the administration of IVIG and the duration of fever. Although this study scored 7 stars on the Newcastle-Ottawa Quality Assessment Scale, the sample size was small (64 participants); this may have had an impact upon the on the accuracy of this research. As platelet counts can be as high as 150,000 to 450,000 platelets per microliter of blood, it is difficult to distinguish a remarkable effect with a 1,000-platelet increase in platelet count. Consequently, we considered that the adoption of an appropriate cut-off point could be helpful, as in previous studies (18, 30, 56). Hence, we cannot deny a potential connection between platelet count and CAA development, and further research is now needed to determine a stable relationship between these two factors. With regards to total bilirubin, only three previous studies have considered the potential role of bilirubin in CAA; these studies yield contradictory findings and therefore there is a lack of good evidence to support a potential relationship between these factors. We were unable to perform meta-analysis for some important continuous variables, such as lymphopenia, neutrophils, and eosinophils. This was because the cut-off point for these factors varied across different studies. In order to study risk factors in a more appropriate manner, it would be more meaningful for future studies to acquire uniform cut-off values for such continuous variables.

### Strengths and Limitations

Our systematic review has several strengths. This is the first systematic review to comprehensively investigate the risk factors for CAA in children with KD. We used a strict search strategy to screen six databases, including PubMed, Embase, Web of Science, Cochrane Library, National Institutes of Health Clinical Trial Databases and OvidMedline. Based on the Newcastle-Ottawa Scale (NOS), 20/21 included studies scored ≥7 stars, suggested high-quality studies. We included a range of publications involving different ethnic populations from across the world to ensure the applicability of our findings and to investigate a wide range of risk factors for CAA. Furthermore, the application of adjusted ORs helped us to avoid the influence of confounding variables, at least in part. We also determined the heterogeneity between the studies included in sub-group analysis and found that apart from IVIG resistance, all other factors showed low levels of heterogeneity. Our sensitivity analysis showed that the sequential omission of a single study did not significantly influence the observed results and the magnitude of effects.

There are some important limitations to this systematic review that need to be considered. First, there are some differences in the criteria used to diagnose CAA. Second, although all of the publications involved in our meta-analysis were of high quality, all of these studies featured data that was acquired retrospectively. Thus, without cautious interpretation, these outcomes could exert a negative impact. Considering the limited number of studies for some factors, the accuracy and validity of the relationship between these factors and CAA may also be questionable. Besides, because of the intrinsic differences in the design of included studies, such as study types, duration of follow-up and so on, potential bias could not be completely ruled out. Finally, in terms of the assessment of publication bias, we were only able to show that there was no apparent publication bias with respect to male gender when using the Egger regression asymmetry test and the Begg test; we did not have a sufficient sample size to carry out similar tests for the other factors.

## Conclusions

Although a number of indicators have been identified to predict the development of CAA development in pediatric KD patients, these studies have never been systematically reviewed. This can reduce the accuracy of prediction and create difficulties in applying these indicators in clinical scenarios. Our present study confirmed that gender, IVIG resistance, IVIG treatment beyond 10 days of onset of symptomsand increased CRP levels are all significant risk factors for CAA. We also identified reliable evidence to support the fact that more number of presenting symptoms is a protective factor against CAA. We recommend that pediatricians should consider these five reliable factors. By more accurate prediction, and earlier preventative treatment for high risk patients, we can expect a reduction in CAA rates. Further research is now needed to investigate the association between CAA and other factors including the interval between KD onset and IVIG administration, platelet count and the duration of fever. Future research should also aim to determine whether echocardiography should be performed more frequently within 1 month of the onset of KD in IVIG-resistant patients.

## Data Availability Statement

All datasets analyzed for this study are included in the manuscript/Supplementary Files.

## Ethics Statement

This article does not feature any studies with human participants or animals that were carried out by the authors. This systematic review and meta-analysis is based on a collection of data retrieved from studies that have already been published. We did not collect individual patient data and did not have direct contact with any of the included patients.

## Author Contributions

FY, JT, and ML designed and conceived the experiments. FY and BP performed the experiments. FY, BP, and HS analyzed the data. FY and BP contributed reagents, materials, and analysis tools. FY, JT, and ML wrote the manuscript.

### Conflict of Interest

The authors declare that the research was conducted in the absence of any commercial or financial relationships that could be construed as a potential conflict of interest.

## Supplementary Material

Checklist S1 The PRISMA Statement.

Checklist S2 Newcastle-Ottawa Quality Assessment Scale for case control studies and cohort studies.

Supporting Figure 1 Pooled odds ratio for CAA by gender (male vs. female, subgroup analysis according to the duration of follow-up).

Supporting Table 1 The Newcastle-Ottawa Quality Assessment Scale for case control studies.

Supporting Table 2 The Newcastle-Ottawa Quality Assessment Scale for cohort studies.

## Abbreviations

CI: Confidence intervals
CAA: Coronary artery abnormality
IVIG: Intravenous immunoglobulin
KD: Kawasaki disease
ORs: Odds ratios
PRISMA: Preferred Reporting Items for Systematic Reviews and Meta-Analyses.

## References

[1] Kawasaki T. [Acute febrile mucocutaneous syndrome with lymphoid involvement with specific desquamation of the fingers and toes in children]. Allergy. (1967) 16:178–222. 6062087

[2] Sánchezmanubens J Bou R Anton J Diagnosis and classification of Kawasaki disease. J Autoimmun. (2014) 48–49:113–7. 10.1016/j.jaut.2014.01.010 24485156

[3] Newburger JW Takahashi M Gerber MA Gewitz MH Tani LY Burns JC. Diagnosis, treatment, and long-term management of Kawasaki disease: a statement for health professionals from the Committee on Rheumatic Fever, Endocarditis and Kawasaki Disease, Council on Cardiovascular Disease in the Young, American Heart Association. Circulation. (2004) 110:2747–71. 10.1161/01.CIR.0000145143.19711.78 15505111

[4] Burns JC Glodé MP. Kawasaki syndrome. Lancet. (2004) 364:533–44. 10.1016/S0140-6736(04)16814-1 15302199

[5] Harada K. Intravenous gamma-globulin treatment in Kawasaki disease. Acta Paediatr. (1991) 33:805–10. 10.1111/j.1442-200X.1991.tb02612.x 1801561

[6] Kobayashi T Inoue Y Takeuchi K Okada Y Tamura K Tomomasa T. Prediction of intravenous immunoglobulin unresponsiveness in patients with Kawasaki disease. Circulation. (2006) 113:2606. 10.1161/CIRCULATIONAHA.105.592865 16735679

[7] Seki M Kobayashi T Kobayashi T Morikawa A Otani T Takeuchi K. External validation of a risk score to predict intravenous immunoglobulin resistance in patients with kawasaki disease. Pediatr Infect Dis J. (2011) 30:145–7. 10.1097/INF.0b013e3181f386db 20802375

[8] Kobayashi T Morikawa A Ikeda K Seki M Shimoyama S Ishii Y. Efficacy of intravenous immunoglobulin combined with prednisolone following resistance to initial intravenous immunoglobulin treatment of acute Kawasaki disease. J Pediatr. (2013) 163:521–6. 10.1016/j.jpeds.2013.01.022 23485027

[9] Berdej-Szczot E Małecka-Tendera E Gawlik T Firek-Pedras M Szydłowski L Gawlik A. Risk factors of immunoglobulin resistance and coronary complications in children with Kawasaki disease. Kardiol Polska. (2017) 75:261–6. 10.5603/KP.a2016.0179 27995598

[10] Ha KS Jang G Lee J Lee K Hong Y Son C. Incomplete clinical manifestation as a risk factor for coronary artery abnormalities in Kawasaki disease: a meta-analysis. Eur J Pediatr. (2013) 172:343–9. 10.1007/s00431-012-1891-5 23229186

[11] Zhao LL Wang YB Suo L. Meta-analysis of the risk factors for coronary artery lesion secondary to Kawasaki disease in Chinese children. Zhonghua Er Ke Za Zhi. (2011) 49:459–67. 21924062

[12] Fu S Gong F Xie C Zhu W Wang W Shen H. S100A12 on circulating endothelial cells surface in children with Kawasaki disease. Pediatr Res. (2010) 68:165. 10.1203/PDR.0b013e3181e67ce8 20461025

[13] Autier P. Risk factors for breast cancer for women aged 40 to 49 years. Ann Intern Med. (2012) 157:529. 10.7326/0003-4819-157-7-201210020-00016 23027325

[14] Higgins JP Thompson SG Deeks JJ Altman DG. Measuring inconsistency in meta-analyses. BMJ. (2003) 327:557–60. 10.1136/bmj.327.7414.557 12958120 PMC192859

[15] Yeo Y Kim T Ha K Jang G Lee J Lee K. Incomplete Kawasaki disease in patients younger than 1 year of age: a possible inherent risk factor. Eur J Pediatr. (2009) 168:157. 10.1007/s00431-008-0722-1 18478263

[16] Hamza HS Raouf WA Zaher AZ Agha HM. Acute Kawasaki disease with emphasis on the echocardiographic profile: a single center experience. Glob Cardiol Sci Pract. (2017) 2017:e201727. 10.21542/gcsp.2017.27 29564348 PMC5856962

[17] Wilder MS Palinkas LA Kao AS Bastian JF Turner CL Burns JC. Delayed diagnosis by physicians contributes to the development of coronary artery aneurysms in children with Kawasaki syndrome. Pediatr Infect Dis J. (2007) 26:256–60. 10.1097/01.inf.0000256783.57041.66 17484225 PMC2868827

[18] Weng KP Hsieh KS Huang SH Ou SF Ma CY Ho TY. Clinical relevance of the risk factors for coronary artery lesions in Kawasaki disease. Kaohsiung J Med Sci. (2012) 28:23–9. 10.1016/j.kjms.2011.09.002 22226058 PMC11916835

[19] Tajima M Shiozawa Y Kagawa J. Early appearance of principal symptoms of kawasaki disease is a risk factor for intravenous immunoglobulin resistance. Pediatr Cardiol. (2015) 36:1159–65. 10.1007/s00246-015-1136-2 25753685

[20] Song D Yeo Y Ha K Jang G Lee J Lee K. Risk factors for Kawasaki disease-associated coronary abnormalities differ depending on age. Eur J Pediatr. (2009) 168:1315–21. 10.1007/s00431-009-0925-0 19159953

[21] Sabharwal T Manlhiot C Benseler SM Tyrrell PN Chahal N Yeung RS. Comparison of factors associated with coronary artery dilation only versus coronary artery aneurysms in patients with Kawasaki disease. Am J Cardiol. (2009) 104:1743–7. 10.1016/j.amjcard.2009.07.062 19962487

[22] Ruan Y Ye B Zhao X. Clinical characteristics of Kawasaki syndrome and the risk factors for coronary artery lesions in China. Pediatr Infect Dis J. (2013) 32:397–402. 10.1097/INF.0b013e31829dd45e 23722531

[23] Qiu H He Y Rong X Ren Y Pan L Chu M. Delayed intravenous immunoglobulin treatment increased the risk of coronary artery lesions in children with Kawasaki disease at different status. Postgraduate Med. (2018) 130:442. 10.1080/00325481.2018.1468712 29745742

[24] Patel A Holman RC Callinan LS Sreenivasan N Schonberger LB Fischer TK. Evaluation of clinical characteristics of Kawasaki syndrome and risk factors for coronary artery abnormalities among children in Denmark. Acta Paediatr. (2013) 102:385–90. 10.1111/apa.12142 23278838

[25] Kim T Choi W Woo CW Choi B Lee J Lee K. Predictive risk factors for coronary artery abnormalities in Kawasaki disease. Eur J Pediatr. (2007) 166:421–5. 10.1007/s00431-006-0251-8 17033807

[26] Kim BY Kim D Kim YH Ryoo E Sun YH Jeon IS. Non-responders to intravenous immunoglobulin and coronary artery dilatation in Kawasaki disease: predictive parameters in Korean children. Korean Circ J. (2016) 46:542–9. 10.4070/kcj.2016.46.4.542 27482264 PMC4965434

[27] Ghelani SJ Kwatra NS Spurney CF. Can coronary artery involvement in Kawasaki disease be predicted? Diagnostics. (2013) 3:232–43. 10.3390/diagnostics3020232 26835677 PMC4665533

[28] Chen JJ Ma XJ Liu F Yan WL Huang MR Huang M. Epidemiologic features of Kawasaki disease in Shanghai from 2008 through 2012. Pediatr Infect Dis J. (2016) 35:7–12. 10.1097/INF.0000000000000914 26372452

[29] Lega JC Bozio A Cimaz R Veyrier M Floret D Ducreux C. Extracoronary echocardiographic findings as predictors of coronary artery lesions in the initial phase of Kawasaki disease. Arch Dis Childhood. (2013) 98:97–102. 10.1136/archdischild-2011-301256 23235890

[30] Boudiaf H Achir M. The clinical profile of Kawasaki disease in Algerian children: a single institution experience. J Trop Pediatr. (2016) 62:139–43. 10.1093/tropej/fmv090 26655639 PMC4886121

[31] Kim MK Song MS Kim GB. Factors predicting resistance to intravenous immunoglobulin treatment and coronary artery lesion in patients with Kawasaki disease: analysis of the Korean nationwide multicenter survey from 2012 to 2014. Korean Circ J. (2018) 48:71–9. 10.4070/kcj.2017.0136 29171205 PMC5764872

[32] Xu H Fu S Wang W Zhang Q Hu J Gao L. Predictive value of red blood cell distribution width for coronary artery lesions in patients with Kawasaki disease. Cardiol Young. (2016) 26:1151–7. 10.1017/S1047951115002140 26435202

[33] Callinan LS Tabnak F Holman RC Maddox RA Kim JJ Schonberger LB. Kawasaki syndrome and factors associated with coronary artery abnormalities in California. Pediatr Infect Dis J. (2012) 31:894–8. 10.1097/INF.0b013e31825c4d7c 22565293

[34] Kim GB Yu JJ Yoon KL Jeong SI Song YH Han JW. Medium- or higher-dose acetylsalicylic acid for acute Kawasaki disease and patient outcomes. J Pediatr. (2016) 184:125. 10.1016/j.jpeds.2016.12.019 28043685

[35] Ichida F Fatica NS Engle MA O'Loughlin JE Klein AA Snyder MS. Coronary artery involvement in Kawasaki syndrome in Manhattan, New York: risk factors and role of aspirin. Pediatrics. (1987) 80:828–35. 3684392

[36] Koren G Lavi S Rose V Rowe R. Kawasaki disease: review of risk factors for coronary aneurysms. J Pediatr. (1986) 108:388–92. 10.1016/S0022-3476(86)80878-2 3950818

[37] Holman RC Curns AT Belay ED Steiner CA Schonberger LB. Kawasaki syndrome hospitalizations in the United States, 1997 and 2000. Pediatrics. (2003) 112:495–501. 10.1542/peds.112.3.495 12949272

[38] Chang RK The incidence of Kawasaki disease in the United States did not increase between 1988 and 1997. Pediatrics. (2003) 111:1124–5. 10.1542/peds.111.5.1124 12728105

[39] Kobayashi T Fuse S Sakamoto N Mikami M Ogawa S Hamaoka K. A new Z score curve of the coronary arterial internal diameter using the lambda-mu-sigma method in a pediatric population. J Am Soc Echocardiogr. (2016) 29:794–801.e29. 10.1016/j.echo.2016.03.017 27288089

[40] Dallaire F Dahdah N. New equations and a critical appraisal of coronary artery Z scores in healthy children. J Am Soc Echocardiogr. (2011) 24:60–74. 10.1016/j.echo.2010.10.004 21074965

[41] Cattalini M Soliani M Caparello MC Cimaz R. Sex differences in pediatric rheumatology. Clin Rev Allergy Immunol. (2019):293–307. 10.1007/s12016-017-8642-3 28849549

[42] Selmi C Brunetta E Raimondo MG Meroni PL. The X chromosome and the sex ratio of autoimmunity. Autoimmun Rev. (2012) 11:A531–7. 10.1016/j.autrev.2011.11.024 22155196

[43] Menasha J Levy B Hirschhorn K Kardon NB. Incidence and spectrum of chromosome abnormalities in spontaneous abortions: new insights from a 12-year study. Genet Med. (2005) 7:251–63. 10.1097/01.GIM.0000160075.96707.04 15834243

[44] Manlhiot C Yeung RS Chahal N McCrindle BW. Intravenous immunoglobulin preparation type: association with outcomes for patients with acute Kawasaki disease. Pediatr Allergy Immunol. (2010) 21:515–21. 10.1111/j.1399-3038.2010.00987.x 20546528

[45] Kobayashi T Saji T Otani T Takeuchi K Nakamura T Arakawa H. Efficacy of immunoglobulin plus prednisolone for prevention of coronary artery abnormalities in severe Kawasaki disease (RAISE study): a randomised, open-label, blinded-endpoints trial. Lancet. (2012) 379:1613. 10.1016/S0140-6736(11)61930-2 22405251

[46] Kim HK Oh J Hong YM Sohn S. Parameters to guide retreatment after initial intravenous immunoglobulin therapy in Kawasaki disease. Korean Circ J. (2011) 41:379–84. 10.4070/kcj.2011.41.7.379 21860639 PMC3152732

[47] Zhang T Yanagawa H Oki I Nakamura Y Yashiro M Ojima T. Factors related to cardiac sequelae of Kawasaki disease. Eur J Pediatr. (1999) 158:694–7. 10.1007/s004310051181 10485297

[48] Yanagawa H Tuohong Z Oki I Nakamura Y Yashiro M Ojima T. Effects of gamma-globulin on the cardiac sequelae of Kawasaki disease. Pediatr Cardiol. (1999) 20:248–51. 10.1007/s002469900458 10368448

[49] Bai L Feng T Yang L Zhang Y Jiang X Liao J. Retrospective analysis of risk factors associated with Kawasaki disease in China. Oncotarget. (2017) 8:54357–63. 10.18632/oncotarget.17530 28903347 PMC5589586

[50] Blake GJ Ridker PM. Novel clinical markers of vascular wall inflammation. Circ Res. (2001) 89:763–71. 10.1161/hh2101.099270 11679405

[51] Ridker PM Pare G Parker A Zee RY Danik JS Buring JE. Loci related to metabolic-syndrome pathways including LEPR, HNF1A, IL6R, and GCKR associate with plasma C-reactive protein: the Women's Genome Health Study. Am J Hum Genet. (2008) 82:1185–92. 10.1016/j.ajhg.2008.03.015 18439548 PMC2427311

[52] Ross R. Atherosclerosis–an inflammatory disease. N Engl J Med. (1999) 340:115. 10.1056/NEJM199901143400207 9887164

[53] Kim JJ Yun SW Yu JJ Yoon KL Lee KY Kil HR. Common variants in the CRP promoter are associated with a high C-reactive protein level in Kawasaki disease. Pediatr Cardiol. (2015) 36:438–44. 10.1007/s00246-014-1032-1 25266886

[54] Chang FY Hwang B Chen SJ Lee PC Meng CC Lu JH. Characteristics of Kawasaki disease in infants younger than six months of age. Pediatr Infect Dis J. (2006) 25:241–4. 10.1097/01.inf.0000202067.50975.90 16511387

[55] Witt MT Minich LL Bohnsack JF Young PC Kawasaki disease: more patients are being diagnosed who do not meet American Heart Association Criteria. Pediatrics. (1999) 104:e10 10.1542/peds.104.1.e10 10390296

[56] Chantasiriwan N Silvilairat S Makonkawkeyoon K Pongprot Y Sittiwangkul R. Predictors of intravenous immunoglobulin resistance and coronary artery aneurysm in patients with Kawasaki disease. Paediatr Int Child Health. (2018) 38:209–12. 10.1080/20469047.2018.1471381 29768976

