# Supplementary figures and images for: Risk Factors of Coronary Artery Abnormality in Children With Kawasaki Disease: A Systematic Review and Meta-Analysis
Source: Front Pediatr. 2019 Sep 26;7:374. doi: 10.3389/fped.2019.00374 (PMC6776089; doi:10.3389/fped.2019.00374)

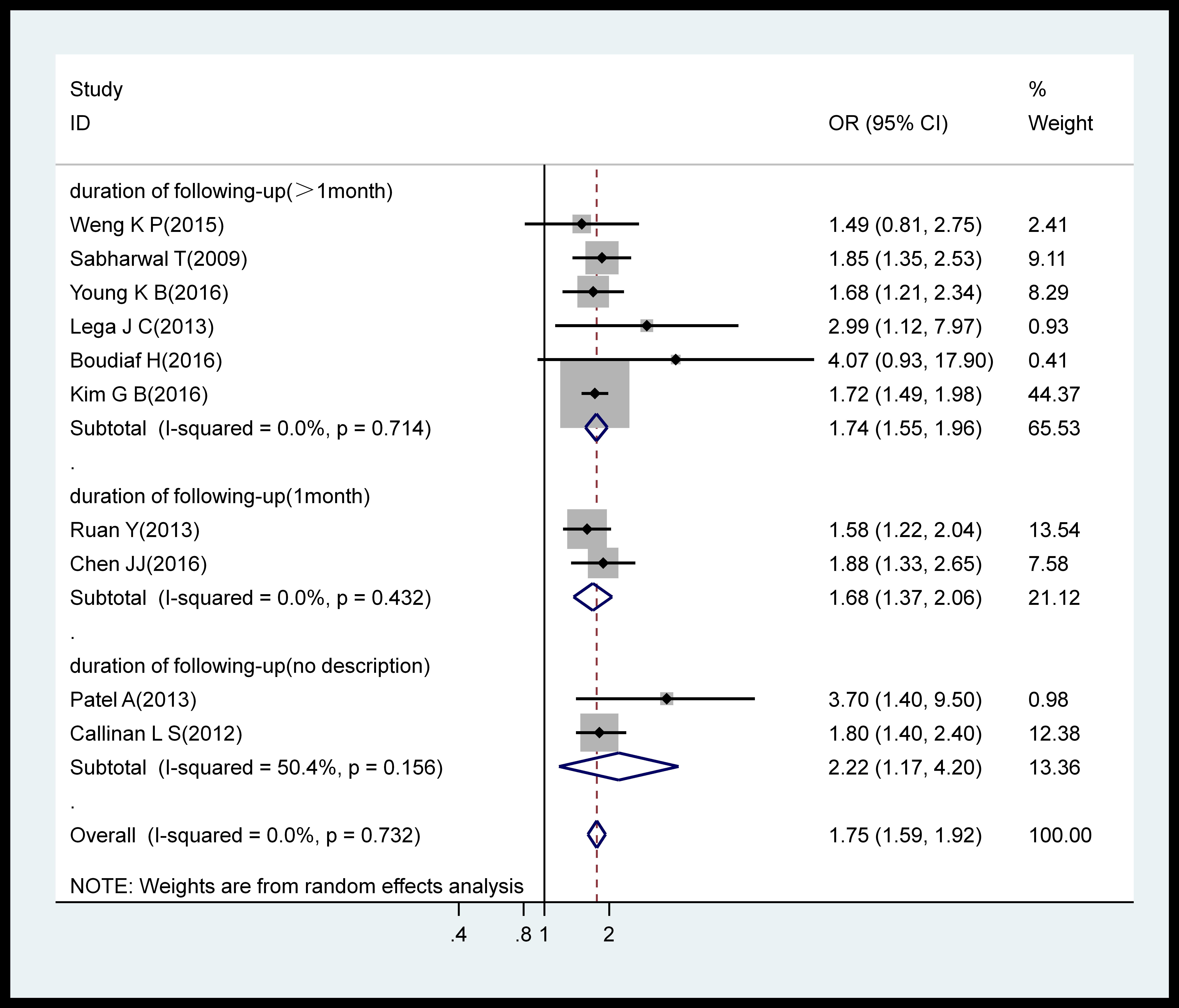

Supplement: Supporting Figure 1 — Pooled odds ratio for CAA by gender (male vs. female, subgroup analysis according to the duration of follow-up). [file Image_1.JPEG]
