# Supplementary material for: Risk Factors of Coronary Artery Abnormality in Children With Kawasaki Disease: A Systematic Review and Meta-Analysis
Source: Front Pediatr. 2019 Sep 26;7:374. doi: 10.3389/fped.2019.00374 (PMC6776089; doi:10.3389/fped.2019.00374)
Supplement: Supporting Table 1 — The Newcastle-Ottawa Quality Assessment Scale for case control studies. [file Table_1.docx]

| First  Author, Year | selection | | | | Comparability | Exposure | | | Total score |
| --- | --- | --- | --- | --- | --- | --- | --- | --- | --- |
|  | Is the case definition adequate | Representativeness of the cases | Selection of Controls | Definition of Controls |  | Ascertainment of exposure | Same method  of ascertainment for cases and controls | Non-Response rate |  |
| Yeo Y,  2010[15] | 1 | 1 | 0 | 1 | 2 | 1 | 1 | 0 | 7 |
| Hamza HS,  2017[16] | 1 | 1 | 0 | 1 | 2 | 1 | 1 | 0 | 7 |
| Wilder MS  2007[17] | 1 | 1 | 0 | 1 | 2 | 1 | 1 | 0 | 7 |
| Tajima M,,  2015[19] | 1 | 1 | 0 | 1 | 2 | 1 | 1 | 0 | 7 |
| Song D I,, 2009[20] | 1 | 1 | 0 | 1 | 2 | 1 | 1 | 0 | 7 |
| Sabharwal T,  2009[21] | 1 | 1 | 0 | 1 | 2 | 1 | 1 | 1 | 8 |
| Ruan Y,  2013[22] | 1 | 1 | 0 | 1 | 2 | 1 | 1 | 0 | 7 |
| Patel A,  2013 [24] | 1 | 1 | 0 | 1 | 2 | 1 | 1 | 0 | 7 |
| Kim T,  2007[25] | 1 | 1 | 0 | 1 | 2 | 1 | 1 | 0 | 7 |
| Ghelani S J,  2013[27] | 1 | 1 | 0 | 1 | 2 | 1 | 1 | 0 | 7 |
| Chen JJ,  2016[28] | 1 | 1 | 0 | 1 | 2 | 1 | 1 | 0 | 7 |
| Lega J C,  2013[29] | 1 | 1 | 0 | 1 | 2 | 1 | 1 | 0 | 7 |
| Boudiaf H,  2016[30] | 1 | 1 | 0 | 1 | 2 | 1 | 1 | 1 | 8 |
| Berdej-Szczot  E,2017[9] | 1 | 1 | 0 | 1 | 2 | 1 | 1 | 1 | 8 |
| Kim MK,  2018[30] | 1 | 1 | 0 | 1 | 2 | 1 | 1 | 0 | 7 |
| Xu H,  2016[32] | 1 | 1 | 0 | 1 | 2 | 1 | 1 | 1 | 8 |
| Callinan L S,  2012[33] | 1 | 1 | 0 | 1 | 2 | 1 | 1 | 0 | 7 |

**Supporting Table1. The NEWCASTLE-OTTAWA SCALE for case control studies.**
