# Supplementary material for: Risk Factors of Coronary Artery Abnormality in Children With Kawasaki Disease: A Systematic Review and Meta-Analysis
Source: Front Pediatr. 2019 Sep 26;7:374. doi: 10.3389/fped.2019.00374 (PMC6776089; doi:10.3389/fped.2019.00374)
Supplement: Supporting Table 2 — The Newcastle-Ottawa Quality Assessment Scale for cohort studies. [file Table_2.docx]

| First Author, Year | selection | | | | Comparability | Outcome | | | Total score |
| --- | --- | --- | --- | --- | --- | --- | --- | --- | --- |
|  | Representativeness of the exposed cohort | Selection of the non-exposed cohort | Ascertainment of exposure | Demonstration that outcome of interest was not present at start of study |  | Assessment of outcome | Was follow  -up long enough for outcomes to occur | Adequacy of follow up of cohorts |  |
| Weng K P,  2015[18] | 1 | 1 | 1 | 0 | 2 | 1 | 1 | 0 | 7 |
| Qiu H,  2018[23] | 1 | 1 | 1 | 0 | 2 | 1 | 1 | 0 | 7 |
| Young K B,  2016[26] | 1 | 1 | 1 | 0 | 2 | 1 | 1 | 1 | 8 |
| Kim G B,  2016[34] | 1 | 1 | 0 | 1 | 2 | 0 | 1 | 0 | 6 |

**Supporting Table2. The NEWCASTLE-OTTAWA SCALE for cohort studies.**
